# An epidemiological investigation of high-risk infants for Respiratory Syncytial Virus infections: a retrospective cohort study

**DOI:** 10.1186/s13052-024-01627-8

**Published:** 2024-03-25

**Authors:** Michela Servadio, Marco Finocchietti, Chiara Vassallo, Riccardo Cipelli, Franca Heiman, Giulia Di Lucchio, Bianca Oresta, Antonio Addis, Valeria Belleudi

**Affiliations:** 1 Department of Epidemiology of the Regional Health Service Lazio, Dipartimento di Epidemiologia del Servizio Sanitario Regionale del Lazio, Rome, Italy; 2 IQVIA Solutions Italy S.r.l., Milan, Italy; 3 AstraZeneca S.p.A. – Medical Department, Milan, Italy

**Keywords:** RSV, CHD, BPD, Prematurity, Healthcare resources utilization, Drug consumption

## Abstract

**Background:**

Respiratory Syncytial Virus (RSV) infections may lead to severe consequences in infants born preterm with breathing problems (such as bronchopulmonary dysplasia (BPD) and respiratory distress syndrome (RDS)) or congenital heart diseases (CHD). Since studies investigating the influence of different gestational age (WGA) and concomitant specific comorbidities on the burden of RSV infections are scarce, the present study aimed to better characterize these high-risk populations in the Italian context.

**Methods:**

This retrospective, longitudinal and record-linkage cohort study involved infants born between 2017 and 2019 in Lazio Region (Italy) and is based on data extracted from administrative databases. Each infant was exclusively included in one of the following cohorts: (1) BPD-RDS (WGA ≤35 with or without CHD) or (2) CHD (without BPD and/or RDS) or (3) Preterm (WGA ≤35 without BPD (and/or RDS) or CHD). Each cohort was followed for 12 months from birth. Information related to sociodemographic at birth, and RSV and Undetermined Respiratory Agents (URA) hospitalizations and drug consumption at follow-up were retrieved and described.

**Results:**

A total of 8,196 infants were selected and classified as 1,084 BPD-RDS, 3,286 CHD and 3,826 Preterm. More than 30% of the BPD-RDS cohort was composed by early preterm infants (WGA ≤ 29) in contrast to the Preterm cohort predominantly constitute by moderate preterm infants (98.2%), while CHD infants were primarily born at term (83.9%). At follow-up, despite the cohorts showed similar proportions of RSV hospitalizations, in BPD-RDS cohort hospitalizations were more frequently severe compared to those occurred in the Preterm cohort (*p*<0.01), in the BPD-RDS cohort was also found the highest proportion of URA hospitalizations (*p*<0.0001). In addition, BPD-RDS infants, compared to those of the remaining cohorts, received more frequently prophylaxis with palivizumab (*p*<0.0001) and were more frequently treated with adrenergics inhalants, and glucocorticoids for systemic use.

**Conclusions:**

The assessment of the study clinical outcomes highlighted that, the demographic and clinical characteristics at birth of the study cohorts influence their level of vulnerability to RSV and URA infections. As such, continuous monitoring of these populations is necessary in order to ensure a timely organization of health care system able to respond to their needs in the future.

**Supplementary Information:**

The online version contains supplementary material available at 10.1186/s13052-024-01627-8.

## Background

The Respiratory Syncytial Virus (RSV) is a common RNA virus belonging to the *Pneumoviridae* family [1, 2]. In young children, RSV is considered the leading cause of lower respiratory tract infections (LRTI) and related illness [3], especially bronchiolitis [4]. RSV epidemic in Italy, as well as in northern hemisphere, typically follows a seasonal pattern that usually starts between October and November, peaks during the winter season (January and February) and resolves between April and May [5]. According to this seasonal trend, on an annual basis, countries organize healthcare services planning [6]. Globally, it has been recently reported that in 2019 there were 3.6 million LRTI RSV-associated hospital admissions in children under 5 years of age, of which 1.4 million were in infants aged 0-6 months, and around 26,300 resulted in death, of which around 50% were infants aged 0-6 months [7]. In Italy, over a 5-year period (2014-2019), 62.5% RSV hospitalization cases were found in infants under 3 months of age and 41.0% in neonates younger than 30 days [8]. Indeed, the severe clinical manifestations of RSV infection are more likely to require hospitalizations in specific high-risk populations, including the elderly (≥ 65 years), adults with chronic heart or lung disease [9, 10] and infants and children up to 24 months of age [11–13]. Furthermore, specific factors are associated with higher risks of RSV hospitalization in infants and children, including prematurity, especially early prematurity, and clinical conditions both in preterm and full-term infants and children (i.e., bronchopulmonary dysplasia (BPD), congenital heart disease (CHD), neurological and neuromuscular diseases) [14]. In particular, in infants and children younger than 24 months, BPD and CHD have been associated with the highest risk of severe symptoms and hospitalization following RSV infection [15–17]. In line with this evidence, the approved therapeutic indication of passive immunoprophylaxis, the only available treatment in Italy against serious RSV infections provided by EMA, is for protection of children and infants at risk of serious LRT diseases following RSV infection [18, 19]. In addition, also respiratory distress syndrome (RDS), a common condition found in preterm infants [20], has been identified as a risk factor for RSV hospitalizations during the first year of life in preterm infants [21, 22].

Healthcare service planning should particularly focus on the most fragile populations and consider the prevalence of high-risk concomitant conditions in these populations. Although association between prematurity and BPD has been extensively demonstrated, as reported by a recent systematic review dedicated to this topic [23], and specific congenital heart diseases in newborns have been found as a relevant risk of developing BPD [24, 25], most of the available studies focusing on characterizations of high-risk population for serious RSV infections separately evaluated the presence of high-risk conditions in infants hospitalized following RSV infections and studies on real-word data on this topic are lacking. A series of systematic reviews revealed that RSV hospitalizations rate in preterm infants and children without BPD or CHD is extremely variable ranging from ~5 to > 100 per 1000 [26], in infants and children with BPD the rate varying from 8.8% to 46.2% [27] and in those with CHD RSV hospitalizations range from 14 to 357 per 1000 [28]. Overall, similarly to what was stated in a recent literature review [29], in order to determine the true burden of RSV infections, further studies should focus on RSV infections and related outcomes in heterogeneous populations with different gestational age and concomitant clinical conditions.

For these reasons, with the present population-based study, we aimed to describe and characterize, in the Italian context, infants that have a high risk of severe RSV infections, using healthcare administrative databases. Specifically, our analyses focus on the socio-demographic characteristics and clinical outcomes, primarily hospitalizations during the first year of life, of high risk infants born between 2017 and 2019 in Lazio Region.

## Methods

### Study design and cohorts

This is a retrospective, longitudinal and record-linkage cohort study. The infants included in the study were those with a gestational age ≤ 35 weeks (WGA ≤ 35), with or without a diagnosis of BPD (and/or RDS), and those with a diagnosis of CHD, born alive in Lazio Region between January 2017 and December 2019. Since RDS, especially in early preterm infants, may evolve into BPD [30] and, as reported in the recently published European Consensus Guidelines on the Management of RDS, the aim of RDS management is to minimize developing of BPD [31], infants with a diagnosis of one or both these two clinical conditions, were included in the same cohort.

Infants were selected using two main administrative databases: the Certificate of Delivery Care (CEDAP) of Lazio Region and Hospital Information System (HIS). CEDAP is a national registry and the primary source of information about newborns, including WGA, birth weight, sex, date of birth and specific information about sociodemographic of parents; HIS collects information on all hospital admissions and discharges registered in regional hospitals, including dates, diagnoses, procedures (primary and secondary) and internal transfer, such as admission to Intensive Care Unit (ICU). Identification of CHD and BPD (and/or RDS) diagnoses was performed through specific International Classification of Diseases, Ninth Revision, Clinical Modification (ICD-9-CM) codes found in hospital discharge records after birth hospitalizations (ICD-9 codes used are available in Table 1S of supplementary materials). According to WGA and comorbidities, infants were categorized into 3 mutually exclusive cohorts with a specific priority criterion: BPD-RDS cohort (priority 1), CHD cohort (priority 2), prematurity (priority 3):

- BPD-RDS cohort: infants with WGA ≤ 35 and a diagnosis of BPD and/or RDS and absence or presence of CHD;
- CHD cohort: infants with CHD and absence or presence of prematurity;
- Preterm cohort: infants with WGA ≤ 35 without BPD (and/or RDS) or CHD.

The present study in accordance with national legislation regarding observational study, was notified to the local ethical committee (Comitato Etico Lazio 1, protocol number 1133).

### Variables and outcomes measures

All infants were observed for a maximum period of 12 months after birth date (index date) and specific outcomes, and related information occurring during this observational period were extracted and analyzed. At birth, specific infants’ characteristics (sex, WGA, birth month, birth weight and duration of birth hospitalization) and mothers’ characteristics (age, education, occupation) were extracted from CEDAP and HIS.

At follow-up specific outcomes related to infants’ hospitalizations were extracted from HIS and ICU and recorded: hospital admission with a diagnosis of RSV infection, hospital admission with a diagnosis of infection caused by an Undetermined Respiratory Agents (URA), number of RSV and URA hospitalizations with Health Care Resources Utilization (HCRU) (i.e., RSV and URA hospitalization with oxygen therapy, or mechanical ventilation, or ICU accesses). In addition, total number of infants receiving first dose of palivizumab (including age at administration), and number of infants receiving first prescription of the following drug categories: antibacterials for systemic use, adrenergics inhalants, selective β-2-adrenoreceptor agonists, glucocorticoids inhalants for obstructive airway diseases and glucocorticoids for systemic use, were also recorded. The exploration on drugs consumption was performed in order to evaluate the utilization of healthcare resources in the cohorts in terms of drugs, during the follow-up period, for the treatment of recurrent wheezing, bronchospasm or childhood asthma. Information about drugs were retrieved from Drug Claims Registry (DCR), a registry collecting information on drug prescriptions reimbursed by the healthcare system, including drugs dispensed by private, public and hospital pharmacies and by local health units. All drugs were identified through the Anatomical Therapeutic Chemical (ATC) classification system (ICD-9 codes and ATC codes used respectively for outcomes and drugs identification, are available in Table 2S and Table 3S of supplementary materials).

### Statistical analysis

Categorial variables were presented as counts and proportions, while continuous variables were presented as mean and standard deviation and median and interquartile range. Cohorts were observed for a follow-up period of 12 months after birth date, RSV and URA-associated hospitalizations and palivizumab administration were counted during RSV seasons (October-April). Proportions refer to the number of infants with at least one event of interest during the follow-up period/total cohort. For each proportion, related to events of interest, was calculated the confidence interval (CI) at 95% and statistical analysis was performed to compare the 3 cohorts, using Pearson's Chi-square Test (Fisher's exact where appropriate). In addition, as supplementary analyses, the investigation of RSV and URA hospitalizations was repeated for each of the individual risk group of the study. Although our observational period pertained 12 months after birth, we extended the period to 24 months after birth in BPD-RDS and CHD cohorts for RSV and URA hospitalizations, and in all cohorts for drug consumption. Finally, in addition to sociodemographic characteristics, also data regarding palivizumab administration have been stratified by gestational age (i.e., ≤29 WGA and 30-35 WGA). A *p*-value < 0.05 was considered as significant and data management and statistical analyses were carried out using Sas software (Sas Enterprise Guide Vers 7.15, SAS Institute Inc., Cary, NC, USA).

## Results

### Characteristics of study cohorts

**Fig. 1:**
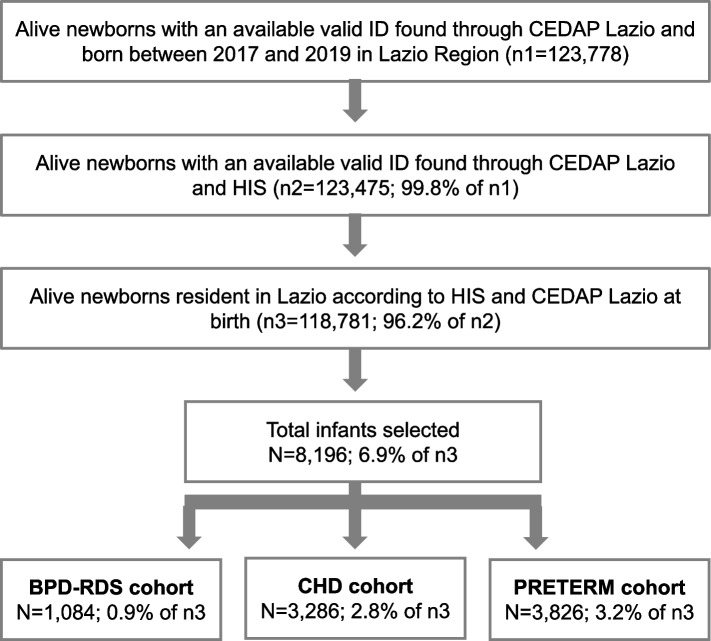
Flowchart of study population *ID* Identification number (Unique anonymous key used for data linkage), *CEDAP* Certificate of Delivery Care, *HIS* Hospital Information System, *BPD* Bronchopulmonary Dysplasia, *RDS* Respiratory Distress Syndrome, *CHD* Congenital Heart Diseases

**Fig. 2:**
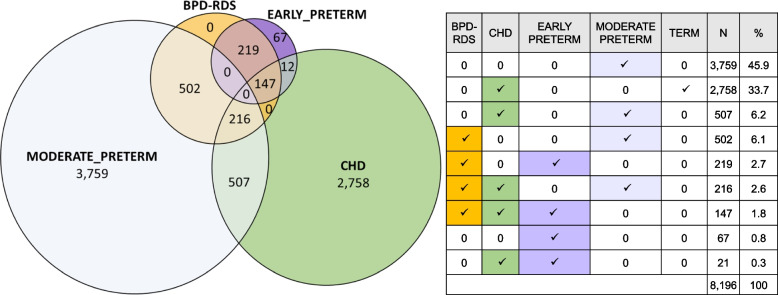
Graphical representation of concomitant risk conditions in the study population Early preterm: infants born at gestational age ≤ 29 weeks; Preterm: infants born at gestational age 30-35 weeks, Term: infants born at gestational age > 35 weeks. *BPD* Bronchopulmonary Dysplasia, *RDS* Respiratory Distress Syndrome, *CHD* Congenital Heart Diseases

Eligible population of the study was composed by infants born between 2017 and 2019 in Lazio Region, resident in this Region at birth and belonging to one of the following mutually exclusive cohorts: infants with BPD and/or RDS, infants born with CHD or preterm infants (WGA ≤35). According to these inclusion criteria, a total of 8,196 infants were selected, of which: 1,084 in the BPD-RDS cohort 3,286 in the CHD cohort and 3,826 in the Preterm cohort. Total number of infants in each cohort corresponded respectively to 0.9%, 2.8% and 3.2% of total infants born in the study period in Lazio Region (Fig. 1). In order to clearly evaluate overlapping clinical conditions among study cohorts, a Venn diagram was implemented (Fig. 2). Distribution of study population according to WGA showed that early prematurity (≤29 WGA) highly overlaps with BPD (and/or RDS) and CHD conditions, compared to moderate prematurity (30-35 WGA). Indeed, only 67 early preterm infants (equal to 14.8% of the total early preterm population of the study and 1.8% of the Preterm cohort) had no other concomitant comorbidities, against 3,759 of moderate preterm (equal to 75.4% of the total moderate preterm population of the study). The CHD cohort was predominantly composed by infants born at term (83.9% of the total cohort). In the BPD-RDS cohort presence of early preterm infants was not negligible: 33.8% of the total cohort, corresponding to 366 infants of which 147 had also a CHD diagnosis (40.2%). Overall, presence of CHD diagnosis in BPD-RDS cohort was found in 363 infants (33.5%) and stratification of the cohort according to WGA, showed that the presence of CHD diagnosis increased as WGA decreased: 40.2, 30.1% of early preterm and moderate preterm BPD-RDS infants.

Sociodemographic characteristics of the cohorts (Table 1) showed that more than 50% of infants in each cohort were males: 54.3% of BPD-RDS, 52.4% of CHD and 53.6% of Preterm cohorts. BPD-RDS cohort was the one with the lowest birth weight (median: 1.5 Kg (1.1-1.9) and mean: 1.6 Kg (±0.6)) and the longest duration of birth hospitalization (median: 41 days (23 –67) and mean: 51 days (±42.4)). Furthermore, early preterm infants within each cohort were those with the longest duration of birth hospitalizations with median values of 73 days (61-92), 48 days (40-73), 33 days (4-53), and mean values of 82.9 days (±40.4), 54.3 days (±27.5), 32.8 days (±28.7) in BPD-RDS, CHD, and Preterm cohorts respectively (Table 1). Based on mothers’ sociodemographic characteristics at the moment of childbirth, the overall median age was similar across cohorts (~ 34.5 years), more than 50.0% were employed, and 40.0% completed secondary education (had a high school diploma).

**Table 1:** Sociodemographic characteristics

	BPD-RDS						CHD								PRETERM					
	≤29 WGA		30-35 WGA		Overall		≤29 WGA		30-35 WGA		>35 WGA		Overall		≤29 WGA		30-35 WGA		Overall	
**N**	366		718		1084		21		507		2758		3286		67		3759		3826	
**Sex**																				
Male	190	51.9%	399	55.6%	589	54.3%	13	61.9%	236	46.5%	1474	53.4%	1723	52.4%	34	50.7%	2015	53.6%	2049	53.6%
Female	176	48.1%	319	44.4%	495	45.7%	8	38.1%	271	53.5%	1284	46.6%	1563	47.6%	33	49.3%	1744	46.4%	1777	46.4%
**Birth month**																				
October-March	169	46.2%	376	52.4%	545	50.3%	7	33.3%	243	47.9%	1351	49.0%	1601	48.7%	35	52.2%	1926	51.2%	1961	51.3%
April-September	197	53.8%	342	47.6%	539	49.7%	14	66.7%	264	52.1%	1407	51.0%	1685	51.3%	32	47.8%	1833	48.8%	1865	48.7%
**Birth weight**																				
Mean	1.0 (±0.3)		1.8 (±0.5)		1.6 (±0.6)		1.4 (±0.7)		2.0 (±0.5)		3.2 (±0.5)		3.0 (±0.7)		1.8 (±0.9)		2.3 (±0.5)		2.3 (±0.5)	
Median	1.0 (0.8-1.2)		1.8 (1.5-2.1)		1.5 (1.1-1.9)		1.3 (1.1-1.4)		2.0 (1.6-2.3)		3.3 (2.9-3.6)		3.2 (2.7-3.5)		1.4 (1.1-2.4)		2.3 (1.9-2.6)		2.2 (1.9-2.6)	
**DBH**																				
Mean	82.9 (±40.4)		34.5 (±33.1)		51 (±42.4)		54.3 (±27.5)		23.5 (±20.8)		9.5 (±18.5)		12 (±19.9)		32.8 (±28.7)		13.8 (±18.1)		14.1 (±18.5)	
Median	73 (61-92)		30 (17- 42)		41 (23-67)		48 (40 - 73)		19 (12 - 30)		5 (4 - 9)		6 (4 - 12)		33 (4 - 53)		10 (6.- 17)		10 (6 - 17)	
**Age** ^a^																				
Mean	35.1 (±6.3)		34.9 (±6.2)		35.0 (±6.3)		34.6 (±5.4)		35.6 (±6.3)		33.8 (±5.8)		34.1 (±5.9)		32.6 (±5.0)		34.4 (±6.4)		34.4 (±6.4)	
Median	35.4 (31.1-39.1)		35.2 (30.8-39.1)		35.4 (31.0-39.1)		35.1 (30.3-37.9)		35.9 (31.4-39.8)		33.9 (30.0-37.8)		34.2 (30.2-38.1)		32.8 (29.0-36.2)		34.6 (30.1-38.8)		34.5 (30.1-38.8)	
**Occupation** ^a^																				
Employed	191	52.2%	388	54.0%	579	53.4%	8	38.1%	339	66.9%	1686	61.1%	2033	61.9%	29	43.3%	1949	51.8%	1978	51.7%
Unemployed	110	30.1%	185	25.8%	295	27.2%	8	38.1%	94	18.5%	444	16.1%	546	16.6%	15	22.4%	961	25.6%	976	25.5%
Housewife	52	14.2%	117	16.3%	169	15.6%	4	19.0%	64	12.6%	472	17.1%	540	16.4%	17	25.4%	667	17.7%	684	17.9%
Other	13	3.6%	28	3.9%	41	3.8%	1	4.8%	10	2.0%	156	5.7%	167	5.1%	6	9.0%	182	4.8%	188	4.9%
**Education** ^a^																				
Low	24	6.6%	46	6.4%	70	6.5%	-	-	29	5.7%	335	12.1%	364	11.1%	10	14.9%	361	9.6%	371	9.7%
Middle	74	20.2%	131	18.2%	205	18.9%	7	33.3%	93	18.3%	504	18.3%	604	18.4%	20	29.9%	832	22.1%	852	22.3%
High	182	49.7%	352	49.0%	534	49.3%	9	42.9%	269	53.1%	1275	46.2%	1553	47.3%	24	35.8%	1621	43.1%	1645	43.0%
Higher	71	19.4%	167	23.3%	238	22.0%	5	23.8%	113	22.3%	594	21.5%	712	21.7%	11	16.4%	866	23.0%	877	22.9%
Missing	15	4.1%	22	3.1%	37	3.4%	-	-	3	0.6%	50	1.8%	53	1k6%	2	3.0%	79	2.1%	81	2.1%

^a^Variables related to mothers. Categorial variables are expressed as counts and proportions, continuous variables are expressed as mean and standard deviation and median and interquartile range. Other occupations include: student, retired/disable, uncertain job and missing values

*BPD* Bronchopulmonary Dysplasia; *RDS* Respiratory Distress Syndrome; *CHD* Congenital Heart Diseases; *WGA* weeks of gestational age; *DBH* duration of birth hospitalization

### RSV and URA hospitalizations at follow-up

The incidence of RSV and URA-associated hospital admissions was assessed during the first year of life and showed that hospitalizations for URA (i.e., undefined pathogen diagnosis) were more frequent in all cohorts compared to hospitalizations with an RSV diagnosis.

**Fig. 3:**
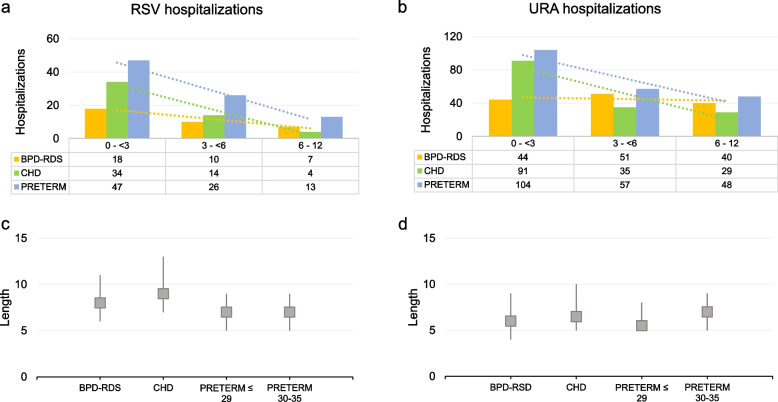
Characteristics of RSV and URA hospitalizations In figure **a** and **b** hospitalizations are expressed as frequency. In figure **c** and **d** duration is expressed in days (median (q1-q3). *BPD* Bronchopulmonary Dysplasia, *RDS* Respiratory Distress Syndrome, *CHD* Congenital Heart Diseases, *RSV* Respiratory Syncytial Virus, *URA* Undetermined Respiratory Agents

Total numbers of infants hospitalized for RSV in BPD-RDS, CHD and Preterm cohorts were respectively 23, 55 and 86, corresponding to 2.1% (95% CI, 1.3-3.0), 1.7% (95% CI, 1.2-2.1) and 2.2% (95% CI, 1.8-2.7) of each cohort (Table 2), whereas values for URA hospitalizations were respectively 102, 162 and 206, corresponding to 9.4% (95% CI, 7.7-11.1), 4.9% (95% CI, 4.2-5.7) and 5.4% (95% CI, 4.7-6.1). No statistically significantly differences were found between RSV hospitalizations among the cohorts, while the higher proportion of URA hospitalizations found in BPD-RDS cohort, compared to those of CHD and Preterm cohorts, was statistically significant (*p* < 0.0001) (Table 2). More infants hospitalized with RSV infection required HCRU (i.e. oxygen therapy, mechanical ventilation, ICU accesses) compared to those hospitalized due to URA infections. As such, 52.2, 34.5 and 20.9% of RSV hospitalizations, respectively found in BPD-RDS, CHD and Preterm cohorts, required HCRU, in contrast to 29.4, 19.1 and 18.4% of URA hospitalizations. In particular, HCRU during RSV and URA hospitalizations was higher for infants in BPD-RDS cohort compared to infants in Preterm cohort (HCRU during RSV: *p* <0.01; HCRU during URA: *p*<0.05) (Table 2). Regarding the analyses repeated for each single risk group represented in Fig. 2, although the small number of events obtained do not support any statistical analyses, results about RSV hospitalizations do not suggest any relevant differences between each group, while early preterm infants with BPD (and/or RDS) and CHD, followed by early preterm infants with BPD (and/or RDS) were those with the highest proportions URA hospitalizations compared to the other risk groups (Table 4S). We also analyzed data about the second year of life for both BPD-RDS and CHD cohorts and observed that RSV and URA hospitalizations, including severe hospitalizations with HCRU occurred less frequently compared to the first year of life, even if proportions of URA and RSV hospitalizations with HCRU (severe hospitalizations) in BPD-RDS cohort remained high (Table 5S). Graphical distribution of frequency of first hospitalization events according to infants’ age and median duration of hospitalizations are represented in Fig. 3. In particular, the vast majority of RSV hospitalizations in each cohort occurred during the first 6 months of life, specifically 80.0 % in BPD-RDS cohort, 92.3% in CHD cohort and 84.9% in Preterm cohort, while a lower proportions of URA hospitalizations occurred during the first 6 months of life, specifically 70.4%, 81.3% and 77.0% in BPD-RDS, CHD and Preterm cohorts respectively (Fig. 3a and b). Median duration of RSV hospitalizations ranged between 7.0 and 8.5 days and in-hospital stay was longer in BPD-RDS and CHD cohorts than in Preterm cohorts (both in early and moderate Preterm sub-cohorts), but no statistically significant differences were found. In terms of length of hospital stay, URA hospitalizations were more similar between cohorts (Fig. 3b and c).

**Table 2:** RSV and URA hospitalizations at first year of follow-up

	BPD-RDS				CHD				PRETERM			
*Outcomes*	*N*	*Proportion*	*95% CI*		*N*	*Proportion*	*95% CI*		*N*	*Proportion*	*95% CI*	
RSV hospitalizations	23	2.1%	1.3	3.0	55	1.7%	1.2	2.1	86	2.2%	1.8	2.7
with HCRU	12	52.2%**	31.8	72.6	19	34.5%	22.0	47.1	18	20.9%	12.3	29.5
URA hospitalizations	102	9.4%***	7.7	11.1	162	4.9%	4.2	5.7	206	5.4%	4.7	6.1
with HCRU	30	29.4%*	20.6	38.3	31	19.1%	13.1	25.2	38	18.4%	13.1	23.7

***p<0.0001 for BPD-RDS cohort versus CHD and Preterm cohorts; **p<0.01 for BPD-RDS versus Preterm cohort; *p<0.05 for BPD-RDS cohort versus Preterm cohort (χ2 test)

*BPD* Bronchopulmonary Dysplasia, *RDS* Respiratory Distress Syndrome, *CHD* Congenital Heart Diseases, *RSV* Respiratory Syncytial Virus, *URA* Undetermined Respiratory Agents, *HCRU* Health Care Resources Utilization, *CI* Confidence Intervals

### Drug consumption at follow-up

By analyzing drug consumption during the first year of life we found that BPD-RDS infants were those more frequently receiving palivizumab. Specifically, during the first RSV season after birth, 48.6% (95% CI, 45.6-51.6) of BPD-RDS cohort was treated with at least one dose of palivizumab, in contrast to 7.2% (95% CI, 6.3-8.0) and 11.0% (95% CI, 10.0%-11.9%) of CHD and Preterm cohorts respectively (Table 3). Stratification of infants that received palivizumab according to age at first dose showed that 31.9% BPD-RDS, 44.7% CHD and 56.8% Preterm infants received prophylaxis at <3 months of age, 40.9% BPD-RDS, 34.0% CHD and 29.1% Preterm infants between 3 and <6 months of age, and 27.2% BPD-RDS, 21.3% CHD and 14.1% Preterm infants between 6 and 12 months of age. Palivizumab administration, within each cohort, was inversely related to WGA and directly related to the number of concomitant clinical conditions. As such, across cohorts, early preterm infants were those treated the most: 78.7% (BPD-RDS), 71.4% (CHD) and 47.8% (Preterm). A similar trend was found also among moderate preterm infants, with the following values: 33.3% in BPD-RDS cohort, 21.5% in CHD cohort and 10.3% in Preterm cohort. According to statistical analyses, among moderate preterm subcohorts, the proportion of BPD-RDS infants treated with palivizumab was significantly higher compared to CHD and Preterm cohorts (*p* < 0.0001). Likewise, drug consumption was higher in BPD-RDS cohort. In particular, statistically significantly higher values were found for adrenergics inhalants (30.4% (95% CI, 27.6-33.1)), where the contribution of selective beta-2 adrenoreceptor agonists account for 17.3% (95% CI, 15.1-19.6) of the total, and glucocorticoids for systemic use (20.8% (95% CI, 18.3-23.2)) (Table 3). Data regarding the second year of life showed that, while in CHD and Preterm cohorts drug consumption decreased compared to the first year of life, in BPD-RDS cohort drug consumption remained high (Table 6S).

**Table 3:** Drug consumption at first year of follow-up

	BPD-RDS				CHD				PRETERM			
*Drugs*	*N*	*Proportion*	*95% CI*		*N*	*Proportion*	*95% CI*		*N*	*Proportion*	*95% CI*	
Palivizumab	527	48.6%**	45.6	51.6	235	7.2%	6.3	8.0	419	11.0%	10.0	11.9
Early preterm	288	78.7%	74.5	82.9	15	71.4%	52.1	90.8	32	47.8%	35.8	59.7
Moderate-late preterm	239	33.3%	29.8	36.7	109	21.5%	17.9	25.1	387	10.3%	9.3	11.3
Born at term	-				111	4.0%	3.3	4.8	-			
Antibacterials for systemic use	426	39.3%	36.4	42.2	1368	41.6%	39.9	43.3	1498	39.2%*	37.6	40.7
Adrenergics. inhalants	329	30.4%**	27.6	33.1	786	23.9%	22.5	25.4	909	23.8%	22.4	25.1
Glucocorticoids for obstructive airway diseases, inhalants	342	31.5%	28.8	34.3	1047	31.9%	30.3	33.5	1114	29.1%*	27.7	30.6
Glucocorticoids for systemic use	225	20.8%**	18.3	23.2	512	15.6%	12.4	18.7	593	15.5%	14.3	16.7

*p<0.05 for Preterm cohort versus CHD cohort; **p<0.001 for BPD-RDS cohort versus CHD and Preterm cohorts (χ2 test)

*BPD* Bronchopulmonary Dysplasia, *RDS* Respiratory Distress Syndrome, *CHD* Congenital Heart Diseases, *CI* Confidence Intervals

## Discussion

The results of the present retrospective cohort study provide an epidemiological description of infants at higher risk for severe RSV infection and related hospitalization in Italy. In particular, descriptive analyses focused on socio-demographics at birth and on specific outcomes registered at follow-up during the first year of life: hospitalizations associated with RSV and URA infections, related HCRU, palivizumab administration and drug consumption by specific categories. According to the present findings, mothers’ socio-demographic characteristics appeared similar among the study cohorts, while infants’ socio-demographic characteristics and study outcomes revealed that different levels of vulnerability exist between the study cohorts. Indeed, presence of clinical conditions, especially BPD and/or RDS, is more frequently associated with severe RSV hospitalizations and URA hospitalizations, palivizumab administration and drug consumption, mainly during the first year of follow-up. The exploration of concomitant risk conditions in our sample, showed that BPD (and/or RDS) diagnosis is more frequently associated with other risk conditions, compared to CHD diagnosis and prematurity, suggesting that the poorer clinical outcomes found in BPD-RDS cohort compared to the other cohorts may be also related to the presence of overlapped clinical and risk conditions.

The prevalence of concomitant BPD (and/or RDS), CHD, early and moderate prematurity was differentially distributed among the cohorts. Specifically, while the Preterm cohort was predominantly composed by moderate preterm infants without concomitant BPD (and/or RDS) or CHD, early prematurity condition highly overlapped with the other risk conditions. As a result, early preterm infants were only 1.8% of the Preterm cohort. In particular, a strong association between early prematurity and BPD (and/or RDS) was found in our study. Indeed, considering the overall number of early preterm infants, about 80.0% (366/454) were in BPD-RDS cohort and about 50.0% (219/454) had only BPD (and/or RDS). These findings are in line with previous studies indicating that incidence of BPD increases as gestational age decreases [32–35]. In particular, Mowitzel and colleagues, using Medicaid databases, found that in a cohort of extremely preterm infants (WGA <28), excluding those with diagnosis of CHD, presence of BPD was found in 61.9% of infants [36]. In previous studies, involving extremely preterm infants with WGA between 22 and 28, BPD diagnosis was performed using clinical database and the resulting percentage was around 50.0% [32, 34]. Despite this value is apparently lower compared to our, percentage rose to 75.0% in infants with WGA 22-24 [34], suggesting that in our sample there might be a huge number of infants born between 22 and 24 WGA, however, since we did not investigate distribution of newborns according to WGA among early preterm infants, this hypothesis is not verifiable. In addition, as already reported in the past [32], different diagnostic criteria used among hospitals, may influence the number of BPD diagnoses, and make comparisons difficult. Moreover, a recent systematic literature review reported a wide range of global BPD incidence in extremely preterm infants, reflecting how different diagnostic criteria and care practices applied across institutions may influence these values [23].

For what concern CHD, our descriptive analyses revealed that this risk condition, in contrast to BPD (and/or RDS), is predominantly found in infants born at term (83.9%), while only 0.6% of infants in CHD cohort were extremely preterm. However, we found a considerable number of infants with CHD among BPD-RDS cohort, especially among preterm infants. These findings are supported by previous works where preterm infants with CHD showed a higher likelihood to have BPD compared to those without CHD [37, 38]. Despite an explanation of the increased frequency of BPD among preterm infants with CHD is still not clear, a literature review focused on preclinical and clinical studies reported that BPD condition is strongly associated with presence of patent ductus arteriosus, one of the most common congenital heart diseases [25]. However, other studies investigating the effect of pharmacological or surgical closure of the patent ductus arteriosus on BPD on set, failed to find a decrease in the incidence of the comorbidity after surgical or pharmacological interventions [39].

Furthermore, we found that BPD-RDS infants were the ones with the lowest birth weight and the longest duration of birth hospitalization, and these results worsened as gestational age decreased. These findings are in line with many previous studies where, in addition to WGA, also low birth weight was found as strong risk factor for developing BPD [40, 41], and prolonged hospitalization stay after birth was found in extremely preterm infants with BPD diagnosis, compared to those without [41, 42].

Our findings regarding sociodemographic information and concomitant risk conditions in study cohorts, supported by previous evidence, suggest that BPD-RDS cohort is the most fragile, however, the contribution of other risk conditions to the higher vulnerability found in this cohort is not negligible, since early prematurity, together with CHD, are strongly associated with BPD (and/or RDS) diagnosis.

As extensively reported in the literature [15, 43–45], the first year of life, corresponding to the first year of follow-up of our population, was a more critical period in terms of RSV hospitalizations compared to the second year. Furthermore, over the first 12 months of life, the first 6 months resulted as even more crucial, since more than 70.0% of RSV hospitalization in each cohort occurred during this period, and this evidence is supported by previous studies [15, 44]. Notably, in the study by Kuhdari and colleagues, where RSV hospitalizations were evaluated during the period 2001-2014 in the overall Italian population, it was estimated that 93.0% of RSV hospitalizations in infants and children between 0 and 2 years, occurred in infants with less than 1 year of age and was calculated a rate of 674/100,000 inhabitants versus 1.5/100,000 inhabitants for infants less than 1 years of age and children between 1 and 4 years of age respectively. Compared to our results, the value obtained was slightly lower, since we obtained a rate of 2/100 infants. However, this discrepancy is understandable because Kuhdari's study included the entire Italian population under 1 year of age, whereas our study was limited to the high-risk population, further confirming how risk conditions can influence clinical outcomes. Future studies examining healthy populations in parallel with high-risk populations may provide more comparable results. Focusing on hospital admission events occurring in each study cohort, the presence comorbidity, especially BPD (and/or RDS) was associated with a higher severity of RSV hospitalizations and a higher frequency and severity of URA hospitalizations. Overall, none of the RSV and URA hospitalizations detected were fatal.

Although RDS, CHD, BPD and prematurity are known factors that increase the risk of severe hospitalizations following RSV infections [21, 46], few studies have examined different levels of risk among these vulnerable populations. Similar to our findings, BPD condition has been associated with HCRU during RSV hospitalization in terms of higher ICU access rate, mechanical ventilation utilization and longer length of hospitalization than those without BPD [17]. Probably, the higher prevalence of early preterm infants in BPD cohort, compared to the others, in addition to the respiratory problems related to the clinical condition itself, drive this greater HCRU during RSV hospitalizations. In the study of Lapcharoensap and colleagues, it has been shown that infants with very low birth weight and BPD, besides to higher HCRU and longer birth hospitalization stay, were also more frequently hospitalized (for any causes) during first year of life, than those without BPD and required greater HCRU, and related costs increased as gestational age decreased [41]. According to our results, also HCRU associated to RSV hospitalizations of CHD infants was not negligible, suggesting that presence of this clinical condition, in the absence of a prevalent prematurity (given the low number of preterm infants in this cohort), may increase severity of RSV infections. Relevant HCRU associated with RSV hospitalizations among CHD infants has emerged also from previous works [47–49].

As regards to palivizumab administration, our results showed that percentages of infants treated in each cohort from 0 to <6 months of age were higher compared to those between 6 and 12 months of age, and this is in line with the well-documented higher impact of RSV infection during the first 6 months of life. However, while most of the CHD and preterm infants received prophylaxis during the first 3 months of life, the majority of BPD-RDS infants received the first dose of prophylaxis between 3 and <6 months of age. This discrepancy is probably due to the longer hospital stay after birth found in this cohort compared to the CHD and Preterm cohorts. Considering the burden of RSV infection over the first 6 months of life, as also confirmed by our analyses, we believe that a timelier distribution of first palivizumab doses during the first months of life, may further improve the benefits produced by prophylaxis. As we aimed to estimate drug consumption, including palivizumab administration, among high-risk infants, we only evaluated total number of infants in each cohort treated with palivizumab during the RSV season, and no specific prophylaxis eligibility criteria for moderate preterm infants without clinical conditions (which can receive palivizumab only if < 6 months old at the start of RSV season) were considered. According to the values obtained, in BPD-RDS cohort palivizumab administration was significantly higher compared to CHD and Preterm cohorts. This finding is in line with the remarkable fragility shown by this cohort in our exploration and with the higher risk of severe RSV hospitalization associated with this diagnosis found in the literature [15, 17]. Palivizumab administration results, stratified by gestational age, showed that early preterm infants of each cohort received more frequently prophylaxis compared to the remaining sub-cohorts and proportions increased with the number of concomitant risk conditions.

However, since we found lower palivizumab administration proportions than expected, we decided to perform an in-depth evaluation of study outcomes (including palivizumab administration, drug consumption and RSV and URA hospitalizations). In particular, in order to have a more defined population that may even more represent the regional health-care management, we repeated same analysis only on infants covered by the regional healthcare system up to 24 months after birth date. While results concerning drug consumption and RSV and URA hospitalizations did not differ from the main results, proportions of palivizumab administration slightly increased by ~ 8.0, 1.0 and 1.5 percentage points in BPD-RDS, CHD and Preterm cohorts respectively, rising to the following values: 56.9%, 8.2% and 12.5%. Similarly, also results stratified by gestational age increased, especially in early preterm sub cohorts where the percentages rose by ~ 10 points increment, reaching these values: 87.2%, 78.9% and 59.3%.

These findings further confirm the greater contribution of concomitant early prematurity and presence of comorbidities, especially BPD (and/or RDS), to the definition of fragility in this population. Defining the vulnerable sub-groups of infants is a crucial aspect for prophylaxis administration strategies and definition of related guidance. In accordance with our finding, a recent study conducted in the USA [36] found that, over a period of 2 years corrected age, 73.1% of extremely preterm infants and children diagnosed with BPD were treated with at least one dose of palivizumab in contrast to 31.9% of those without, these percentages are similar to ours, where 78.7% of early preterm infants with BPD (and/or RDS) and 47.8% of those without received administration of palivizumab. The slightly higher percentages found in our study may be partially related to different gestational age considered, indeed while in the study of Mowitz and colleagues, extreme prematurity indicates WGA <28, in our study early preterm infants have WGA ≤29. In a further study, investigating palivizumab administration in infants and children at high risk of RSV complications, was reported that infants with chronic lung disease, or extreme prematurity (WGA <28) were more likely to be treated with palivizumab [50]. Moreover, in line with our findings, several studies indicated that infants with diagnosis of BPD are more likely to receive palivizumab compared to those with diagnosis of CHD [50–52].

In accordance with their higher fragility, BPD-RDS infants were also those more frequently exposed to the following drug categories: adrenergics  inhalants, and glucocorticoids for systemic use. Although previous investigations of drug consumption in this population are scarce, the higher drug consumption found in this cohort can be related to the higher presence of extremely preterm infants, as well as the greater respiratory problems related to the diagnosis itself. In a population-based study analyzing drug prescriptions in preterm children (WGA < 37) was reported that these children had higher risk of being prescribed/dispensed with anti-asthmatic drugs (beta-2 agonists and glucocorticoids) compared to full-term children [53]. In addition, a recent study has demonstrated that the use of inhaled bronchodilators and systemic corticosteroids is higher in extremely preterm infants with BPD compared to those without [36], and these findings are in line with our findings, considering the prevalence of early prematurity in BPD-RDS cohort.

### Strengths

Our study has several strengths. To the best of our knowledge this is one of the few studies in Italy investigating from an epidemiological point of view the populations at highest risk for severe RSV infections and prevalence of concomitant risk conditions. Our descriptive results suggest that early prematurity is strongly associated with BPD (and/or RDS) condition and that preterm infants with CHD seem to be more frequently associated with BPD (and/or RDS) compared to those born at term. This evidence provides important information concerning the level of fragility of different high-risk populations. In addition, it is one of the few studies that broadly investigates drug consumption in these populations from birth to 24 months. Through the integration of several administrative database, this real word study describes from different point of views a very frail population of infants born in Lazio Region, which represents ~ 10.0% of Italian population with these characteristics.

Furthermore, an additional strength of our study is to have highlighted the importance of monitoring the impact of RSV epidemic in a population that, in the future, would be even more affected by the influence of environmental factors related to climate change [54, 55] and concomitant circulation of new viruses, like SARS-CoV-2 [56, 57], on the incidence and severity of RSV and other viral respiratory pathogens. In this context, it is important to mention the consequences of the significant change in RSV seasonality registered during and after the COVID-19 pandemic, which produced first a strong reduction in RSV transmission and a subsequent unexpected increase of RSV-associated hospitalizations starting from late spring 2021 and during the summer and autumn months [58–61]. In Italy admissions for bronchiolitis sharply increased between September and November 2021, by four folds as compared to pre-pandemic year 2019 [57].

### Limitations

Our study presents some limitations that are mostly related to the source of the data. Indeed, our analyses are based on data retrieved from administrative database, where several clinical characteristics and information are not available. For instance, in Italy, where the molecular diagnosis of infectious agents is not routinely done during hospitalization and an RSV surveillance system, similar to the one present in other countries, has not been implemented yet, many cases of hospitalization are probably classified as URA, instead of RSV. As a consequence, in the present study there might be an underestimation of RSV hospitalizations, even if the extraction of both RSV and URA hospitalizations gave us the possibility to distinguish RSV from other respiratory tract infections. In addition, the analysis is based on data from one single Italian Region and results obtained may not reflect trends about similar outcomes of other geographical area. However, Lazio is one of the most populated regions in Italy with around 40,000 newborns per year. Furthermore, the use of ICD-9 codes to identify diagnosis from hospital discharge records, may has approximated actual diagnoses and may has not captured all relevant medical diagnoses, especially for BPD. Being this a study that adopts administrative data from secondary care (excluding drug prescriptions which were analyzed from both primary as well as secondary setting), a limitation of the analysis is the lack of data which are not recorded in such data sources, in particular outpatient data regarding medical visits for respiratory infections. Lastly, possible resolution of CHD (especially the presence of patent ductus arteriosus in CHD cohort) or BPD (and/or RDS) comorbidities after birth were not detectable and as a consequence, this aspect may have overestimated the persistence of comorbidities after birth in the cohorts

## Conclusions

We studied 8,196 infants from diverse fragile populations born in Lazio Region and gathered relevant data for clinicians and decision-makers in Italy and other developed countries.

Altogether, the results of our study showed that there is a need of continuous monitoring of populations at higher risk of developing severe RSV infections. In particular, the different level of fragility found among high-risk infants, especially BPD-RDS and early preterm infants, indicate that these populations would benefit from a more adequate health care management. The future involvement of clinicians with expertise in neonatology in our studies may further improve knowledge on this topic and better clarify evidence regarding concomitant clinical conditions. Indeed, despite our study showed that there is a certain burden of RSV infections also in CHD cohort compared to Preterm cohort, small number of works were dedicated to this comorbidity compared to BPD. Furthermore, given the present and future variations in seasonal trends of RSV epidemic, studies investigating the impact of these variations on clinical outcomes in these at-risk populations are warranted. In this context, studies based on administrative database regarding an entire Region or Country may provide exhaustive findings in a relatively short time, compared to monocentric or multicentric studies. These future perspectives are necessary in order to ensure a timely organization of the healthcare system able to respond to the needs of infants in high-risk populations.

### Supplementary Information

**Supplementary Material 1.**

## Data Availability

The data supporting the findings of this article are available at aggregated level from the authors upon reasonable request and with permission of Lazio region. Requests to access should be directed to corresponding author.
