# Supplementary material for: An epidemiological investigation of high-risk infants for Respiratory Syncytial Virus infections: a retrospective cohort study
Source: Ital J Pediatr. 2024 Mar 25;50:56. doi: 10.1186/s13052-024-01627-8 (PMC10962102; doi:10.1186/s13052-024-01627-8)
Supplement: Supplementary file 1 — Supplementary Material 1. [file 13052_2024_1627_MOESM1_ESM.docx]

**Supplementary Materials**

| **Table 1S. Diagnosis ICD-9 codes used for identification of BPD-RDS and CHD cohorts** | |
| --- | --- |
| *BPD-RDS (Cohort 1)* | *ICD-9-CM code* |
| Chronic respiratory disease arising in the perinatal period | 770.7 |
| Respiratory distress syndrome in newborn | 769 |
| *CHD (Cohort 2)* | *ICD-9-CM code* |
| Congenital heart diseases | 745*; 746*; 747.0-747.4 |
| Note. * Includes all diagnostic subcodes. Abbreviations. BPD: Bronchopulmonary Dysplasia; RDS: Respiratory Distress Syndrome; CHD: Congenital Heart Diseases; ICD-9-CM: International Classification of Diseases, Ninth Revision, Clinical Modification. | |

| **Table 2S. Diagnosis ICD-9 codes used for identification of RSV, URA hospitalizations and related HCRU** | |
| --- | --- |
| *RSV hospitalization* | *ICD-9-CM code* |
| Respiratory syncytial virus Infection | 079.6 |
| Pneumonia due to respiratory syncytial virus | 480.1 |
| Bronchiolitis due to respiratory syncytial virus | 466.11 |
| *URA hospitalization* | *ICD-9-CM code* |
| Acute bronchitis and bronchiolitis | 466^$^ |
| Bronchitis (unspecified if chronic or acute) | 490.1 |
| Viral pneumonia | 480^§^ |
| Bronchopneumonia, organism unspecified | 485 |
| Pneumonia, organism unspecified | 486 |
| *HCRU* | *ICD-9-CM code* |
| Non-invasive mechanical ventilation | 93.90 |
| Intermittent positive pressure breathing [IPPB] | 93.91 |
| Other continuous invasive mechanical ventilation | 96.7* |
| Ventilatory support (mechanical ventilation and/or continuous positive airway pressure [CPAP] ventilators) | 96.01; 96.05; 96.7* |
| Note. ^$^Includes 466.0, 466.1 and 466.19; ^§^Includes 480.8 and 480.9; *Includes all procedures codes.  Abbreviations. RSV: Respiratory Syncytial Virus; URA: Undetermined Respiratory Agents; HCRU: Health Care Resources Utilization; ICD-9-CM: International Classification of Diseases, Ninth Revision, Clinical Modification. | |

| **Table 3S. ATC codes used for identification drugs** | | | |
| --- | --- | --- | --- |
| *Drugs* | *ATC code* | | |
| Palivizumab | J06BB16 |  |  |
| Antibacterials for systemic use | J01 | | |
| Adrenergics, inhalants | R03A | | |
| Selective beta-2-adrenoreceptor agonists | R03AC | | |
| Glucocorticoids for obstructive airway diseases, inhalants | R03BA | | |
| Glucocorticoids for systemic use | H02AB | | |
| Abbreviations. ATC: Anatomical Therapeutic Chemical. | | | |

| **Table 4S. RSV and URA hospitalizations stratified by risk groups** | | | | |
| --- | --- | --- | --- | --- |
| *RSV hospitalizations* | *N* | *Proportion* | *95% CI* | |
| Moderate-late preterm | 84 | 2.2% | 1.8 | 2.7 |
| CHD born at term | 44 | 1.6% | 1.1 | 2.1 |
| CHD moderate-late pretem | 11 | 2.2% | 0.9 | 3.4 |
| BPD-RDS moderate-late preterm | 14 | 2.8% | 1.3 | 4.2 |
| BPD-RDS early preterm | 3 | 1.4% | 0.0 | 2.9 |
| BPD-RDS + CHD moderate-late preterm | 4 | 1.9% | 0.1 | 3.6 |
| BPD-RDS + CHD early preterm | 2 | 1.4% | 0.0 | 3.2 |
| Early preterm | 2 | 3.0% | 0.0 | 7.1 |
| CHD early preterm | 0 | - | - | - |
| *URA hospitalizations* | *N* | *Proportion* | *95% CI* | |
| Moderate-late preterm | 201 | 5.3% | 4.6 | 6.1 |
| CHD born at term | 129 | 4.7% | 3.9 | 5.5 |
| CHD moderate-late preterm | 32 | 6.3% | 4.2 | 8.4 |
| BPD-RDS moderate-late preterm | 41 | 8.2% | 5.8 | 10.6 |
| BPD-RDS early preterm | 22 | 10.0% | 6.1 | 14.0 |
| BPD-RDS + CHD moderate-late preterm | 17 | 7.9% | 4.3 | 11.5 |
| BPD-RDS + CHD early preterm | 22 | 15.0% | 9.2 | 20.7 |
| Early preterm | 5 | 7.5% | 1.2 | 13.8 |
| CHD early preterm | 1 | 4.8% | 0.0 | 13.9 |

Abbreviations. BPD: Bronchopulmonary Dysplasia; RDS: Respiratory Distress Syndrome; CHD: Congenital Heart Diseases; RSV: Respiratory Syncytial Virus; URA: Undetermined Respiratory Agents; HCRU: Health Care Resources Utilization; CI: Confidence Intervals.

| **Table 5S. RSV and URA hospitalizations at second year of follow-up** |
| --- |

|  | BPD-RDS | | | | CHD | | | | PRETERM | | | |
| --- | --- | --- | --- | --- | --- | --- | --- | --- | --- | --- | --- | --- |
| *Outcomes* | *N* | *Proportion* | *95% CI* | | *N* | *Proportion* | *95% CI* | | *N* | *Proportion* | *95% CI* | |
| RSV hospitalizations | 3 | 0.3% | 0.0 | 0.6 | 2 | 0.1% | 0.0 | 0.1 | - |  |  |  |
| with HCRU | 1 | 33.3% | 30.5 | 36.1 | 0 |  |  |  | - |  |  |  |
| URA hospitalizations | 13 | 1.2% | 0.6 | 1.8 | 21 | 0.6% | 0.4 | 0.9 | - |  |  |  |
| with HCRU | 4 | 30.8% | 28.0 | 33.5 | 0 |  |  |  | - |  |  |  |
| Abbreviations. BPD: Bronchopulmonary Dysplasia; RDS: Respiratory Distress Syndrome; CHD: Congenital Heart Diseases; RSV: Respiratory Syncytial Virus; URA: Undetermined Respiratory Agents; HCRU: Health Care Resources Utilization; CI: Confidence Intervals. | | | | | | | | | | | | |

| **Table 6S. Drug consumption at second year of follow-up** | | | | | | | | | | | | |
| --- | --- | --- | --- | --- | --- | --- | --- | --- | --- | --- | --- | --- |
|  | BPD-RDS | | | | CHD | | | | PRETERM | | | |
| *Drugs* | *N* | *Proportion* | *95% CI* | | *N* | *Proportion* | *95% CI* | | *N* | *Proportion* | *95% CI* | |
| Antibacterials for systemic use | 528 | 48.7% | 45.7 | 51.7 | 1559 | 47.4% | 45.7 | 49.2 | 1721 | 45.0%* | 43.4 | 46.6 |
| Adrenergics. inhalants | 295 | 27.2%*** | 24.6 | 29.9 | 594 | 18.1% | 16.8 | 19.4 | 704 | 18.4% | 17.2 | 19.6 |
| Glucocorticoids for obstructive airway diseases, inhalants | 356 | 32.8%** | 30.0 | 35.6 | 913 | 27.8% | 26.3 | 29.3 | 998 | 26.1% | 24.7 | 27.5 |
| [Glucocorticoids for systemic use](https://www.whocc.no/atc_ddd_index/?code=H02A&showdescription=no) | 217 | 20.0%*** | 17.6 | 22.4 | 456 | 13.9% | 12.7 | 15.1 | 554 | 14.5% | 13.4 | 15.6 |
| *P<0.05 for Preterm cohort versus CHD and BPD-RDS cohorts; **P<0.001 for BPD-RDS cohort versus CHD and Preterm cohorts; ***P<0.0001 for BPD-RDS cohort versus CHD and Preterm cohorts.  Abbreviations. BPD: Bronchopulmonary Dysplasia; RDS: Respiratory Distress Syndrome; CHD: Congenital Heart Diseases; CI: Confidence Intervals. | | | | | | | | | | | | |
|  |  |  |  |  |  |  |  |  |  |  |  |  |
